# Alpha Lipoic Acid: A Therapeutic Strategy that Tend to Limit the Action of Free Radicals in Transplantation

**DOI:** 10.3390/ijms19010102

**Published:** 2018-01-04

**Authors:** Nella Ambrosi, Diego Guerrieri, Fiorella Caro, Francisco Sanchez, Geraldine Haeublein, Domingo Casadei, Claudio Incardona, Eduardo Chuluyan

**Affiliations:** 1 CEFYBO-CONICET, Facultad de Medicina, Universidad de Buenos Aires, Buenos Aires C1199ABB, Argentina; nella_ambrosi@hotmail.com (N.A.); fiorellacaro86@gmail.com (F.C.); Franciscosanchezinv@hotmail.com (F.S.); geraldine.vet@gmail.com (G.H.); echuluyan@gmail.com (E.C.); 2 Instituto de Nefrología de Buenos Aires, Nephrology, Buenos Aires C1199ABB, Argentina; drdomingocasadei@gmail.com; 3 GADOR SA, Buenos Aires C1199ABB, Argentina; incardona@gador.com.ar; 4 Hospital Italiano, Buenos Aires C1199ABB, Argentina

**Keywords:** IRI, ROS, α-lipoic acid, transplantation

## Abstract

Organ replacement is an option to mitigate irreversible organ damage. This procedure has achieved a considerable degree of acceptance. However, several factors significantly limit its effectiveness. Among them, the initial inflammatory graft reaction due to ischemia-reperfusion injury (IRI) has a fundamental influence on the short and long term organ function. The reactive oxygen species (ROS) produced during the IRI actively participates in these adverse events. Therapeutic strategies that tend to limit the action of free radicals could result in beneficial effects in transplantation outcome. Accordingly, the anti-oxidant α-lipoic acid (ALA) have been proved to be protective in several animal experimental models and humans. In a clinical trial, ALA was found to decrease hepatic IRI after hepatic occlusion and resection. Furthermore, the treatment of cadaveric donor and recipient with ALA had a protective effect in the short-term outcome in simultaneous kidney and pancreas transplanted patients. These studies support ALA as a drug to mitigate the damage caused by IRI and reinforce the knowledge about the deleterious consequences of ROS on graft injury in transplantation. The goal of this review is to overview the current knowledge about ROS in transplantation and the use of ALA to mitigate it.

## 1. Introduction

During the transplantation procedure, the organs undergo ischemia-reperfusion injury (IRI). This is an inevitable pathological condition characterized by the initial restriction of blood supply followed by subsequent restoration of perfusion with concomitant re-oxygenation of the graft. Injury begins with anoxia, continues and is aggravated by reperfusion of the organ, culminating with a sterile inflammatory reaction [1]. Due to ischemia, structural and metabolic changes occur in tissue such as reduction of capillary diameter, metabolic dysfunction of endothelial cells, malfunction of the cell membrane and the deregulation of inflammatory mediators [2]. Once blood flow is restored, a number of molecular mechanisms are triggered leading to tissue damage and cells death.

In the ischemic phase, anoxic injury begins with a decrease in mitochondrial energy production and, therefore a downfall in adenosine triphosphate (ATP). Due to energy deficiency, imbalances of the cellular ions take place, also activation of hydrolases and a critical increase in the permeability of the cells membrane [3,4,5,6]. These events follow only in part a sequential order and self-amplification of processes and propagation can occur through various pathways. Cytosolic pH decreases due to ATP degradation, increased glycolytic rate with lactate accumulation and the release of H^+^ from damaged lysosomes. In parallel, cellular homeostasis of ions deteriorates, implying an increase in the cytosolic concentrations of Na^+^ and Ca^2+^. The latter activates hydrolases, such as phospholipase A2 and proteases [3] and proteolysis of cytoskeletal proteins favors the process of tissue injury. At the same time, elevated cytosolic Ca^2+^ and hypoxia generate an increase in mitochondrial membrane permeability. In turn, swelling of mitochondria and augmented permeability lead to the release of cytochrome c which activates a signaling pathway involving caspases 1 and 9, that promotes cellular apoptosis. On the other hand, the rise of cellular Na^+^ cause edema, that contributes to the damage of the plasma membrane resulting finally in the cell death by necrosis [7].

Paradoxically, restoration of blood flow initiates a cascade of events that lead to additional cell damage, beyond that caused by ischemia. During re-oxygenation, new lesions are generated by the increase in the production of reactive oxygen species (ROS) by the epithelial and endothelial cells, platelet and activated leukocytes that infiltrate the area [8,9]. These free radicals; such as superoxide anion, hydrogen peroxide and hydroxyl radical; are generated in the reperfused tissues as a consequence of mitochondrial lesions, by an incomplete reduction of oxygen or through the action of oxidases. Under normal conditions, the harmful effects of superoxide are prevented by superoxide dismutase, which converts the anion to hydrogen peroxide, glutathione peroxidase and catalase converts hydrogen peroxide to water [10,11]. During reperfusion, this natural defense is overcome and the hydrogen peroxide is converted into hydroxyl radicals, capable of damaging a wide variety of molecules leading to cell dysfunction or death due to necrosis or apoptosis [12,13,14,15]. The process perpetuates through the release of proinflammatory cytokines which increases the inflammatory response and injury.

## 2. Pharmacological Treatments for IRI Prevention

There are several experimental studies focused both on inhibiting the deleterious effects of ischemia and reperfusion as well as those generated by the inflammatory response. For this purpose, drugs such as chloroquine [16] or chlorpromazine [17], were used to prevent mitochondrial dysfunctions and phospholipid degradation during ischemia. There are also studies that investigated blocking neutrophil activation and infiltration or tumor necrosis factor (TNF)-α proinflammatory cytokine, with specific monoclonal antibodies [18,19]. Also, it was sought to decrease apoptosis by blocking calcium with an antagonist [20]. However, based on the role of ROS in the pathophysiology of IRI, one of the main target to prevent injury should be against the production of ROS.

**Figure 1:**
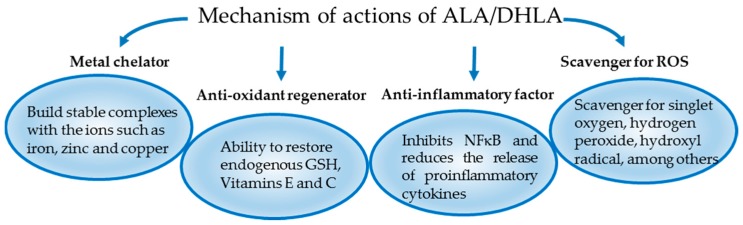
The proposed pleiotropic mechanisms of action of ALA/DHLA.

Several antioxidant drugs may be available to be tested in different IRI models. However, one of the most effective antioxidants used in daily clinical practice is α-lipoic acid (ALA) [21]. It is a powerful natural antioxidant produced in mitochondria from octanoic acid. It has activity in both aqueous and lipid media. It acts both at intra- and extracellular levels and has two isomeric forms. Due to these properties it has a wide potential of pharmacological action [22,23]. Its major biological role is as a cofactor of mitochondrial enzymes such as α-ketoglutarate dehydrogenase and pyruvate dehydrogenase [24]. ALA also appears to be involved in the production of acetyl-coenzyme A (CoA), through the oxidative decarboxylation of pyruvate [25]. In vivo, ALA can be reduced in dihydrolipoic acid (DHLA) which has a higher antioxidant action. Both ALA and DHLA neutralize ROS and have metal chelating capacity for Fe^2+^, Cu^2+^ and Cd^2+^. It has been shown that only DHLA is able to regenerate endogenous antioxidants (glutathione and vitamin E, C) and repair the tissue damage generated by ROS [26]. However, not all of the effects of ALA are due to its anti-oxidant activity. Lipopolysaccharides (LPS) induce proinflammatory cytokines by promoting the phosphorylation of the inhibitor of Nuclear factor-κB alpha (IκBα) and the translocation of NF-κB to the nucleus. It was described that ALA can inhibit the release of proinflammatory cytokines induced by LPS [27]. This anti-inflammatory activity is mediated by the inhibition of phosphorylation of IκBα and the translocation of NF-κB to the nucleus [28]. Moreover, a recent study shows, in a macrophage cell line, that ALA inhibits extracellular signal-regulated kinase (ERK), mitogen-activated protein kinase 14 and NF-κB activation induced by extracellular histones [29]. Therefore, the beneficial effect of ALA, or its reduced form, DHLA, is mediated through different mechanisms of action depicted in Figure 1. It is important to mention that safe levels for oral ALA intake have been defined in rats and 2000 mg/kg is the LD50 for intravenous (i.v.) administration [30].

The wide antioxidant activities of ALA have supported to test the use ALA in several experimental models of IRI in different organs and systems, some of which are described below and in Table 1.

### 2.1. ALA in Nervous System

Ischemic-reperfusion injury in nervous system occurs in conditions such as stroke, subarachnoid hemorrhage or head trauma. The ischemic injury to peripheral nerve can be aggravated by reperfusion, resulting in axon degeneration. Mitsui et al. studied IRI in a sciatic-tibial nerve rat model. The experimental design took into account two groups with different periods of ischemia (3 h or 5 h) but same reperfusion time and ALA treatment. Remarkably, distal sensory conduction was significantly improved and axon degeneration decreased, in the short-time ischemia group treated with ALA, but failed to show favorable effects if the duration of oxygen deprivation was longer. These results suggest that the time of ischemia may be important to observe effects of ALA in IRI (Table 1) [31].

Also, it is important to highlight that, ALA effect was also studied in cerebral IRI. As a result, Panigrahi et al. described a dramatically reduction in mortality rate in animals treated with ALA [39].

### 2.2. ALA in Reproductive System

Torsion of the adnexa is a rare cause of lower abdominal pain and a surgical emergency of difficult diagnosis with a prevalence of 2.7% [40]. In most cases, it is associated with the presence of a preexisting adnexal tumor or cyst, but it can also occur in normal ovaries. Because of the torsion, the ovaries suffer IRI. Cosar et al. showed that pathological changes induced by IRI were reduced in ALA-treated rats; specially the neutrophils infiltration, edema and loss of cohesion in the ovaries. Also, levels of malondialdehyde (MDA), as an index of lipid peroxidation, was significantly decreased by ALA treatment in ovarian tissue and in serum. Finally, it has shown a regulatory activity for superoxide dismutase (SOD), xanthine oxidase (XO) and nitric oxide (NO) serum levels in treated animals [32].

In male, testicular torsion is also a urologic emergency occurring frequently in neonatal and adolescent periods. The testis is sensitive to IRI, which results in testicular cell damage and apoptosis. Ozbal et al. studied ALA effects in testicular IRI, with a model of testicular torsion and detorsion. As a result, pretreatment with ALA reduced cell damage and decreased cell death. Like in the ovaries, ALA decreased MDA tissue levels. However, in the testis IRI model it was also possible to observe greater activity of the enzyme SOD in testicular tissue in animals treated with ALA. This may be related to the different administration protocol chosen in both studies. Nevertheless, both studies showed that ALA pretreatment has beneficial effects in ovarian and testicular IRI models [33].

### 2.3. ALA in Liver

In liver resection and transplantation, IRI is one of the main causes of organ non-function. It is important to emphasis that animals do not tolerate total hepatic ischemia very well and therefore, most hepatic IRI models are partial or ex vivo. As an example, Müller et al. studied the administration of ALA in an ex vivo model. Rat livers were perfused with Krebs–Henseleit buffer with or without ALA, followed by warm ischemia (1 h) and reperfusion (90 min). The preconditioning with ALA significantly reduced lactate deshidrogenase (LDH) and purine nucleoside phosphorylase (PNP) efflux during reperfusion in isolated perfused livers. Post-ischemic activation of NF-κB and activating protein 1 (AP-1) was significantly reduced in ALA-pretreated organs. Then, they used an animal model of hepatic IRI and detected that the preconditioning with ALA reduced glutathione s-transferase (GST) plasma levels and improved liver histology compared to control group. It is important to highlight that this study showed a causal relationship between protein kinase B (Akt) activation and hepatoprotection by ALA. Through this study, it was possible to conclude that the phosphatidylinositol-3-kinases (PI3K)/Akt pathway plays a central protective role in IRI of the rat liver and that ALA administration attenuates IRI via this pathway (Table 1) [34].

It is known that liver is an organ with high regenerative capacity. Duenschede et al. studied ALA effects on liver IRI and regeneration. To assess the effect of ALA in liver IRI, the authors induced 90 min of ischemia of one liver lobe followed by 1 h of reperfusion [41]. As a result, they observed that caspase 3, 8, and 9 activities were significantly lower in the ALA-treated group accompanied by a decrease in DNA fragmentation in hepatocytes. Furthermore, they discovered that ALA had effects on liver regeneration. This was studied by resecting the 70% of non-ischemic liver after ischemia, before reperfusion and analyzing the remaining tissue in untreated and ALA-treated animals. Untreated animals showed massive mitochondrial damage compared with ALA-treated animals. Remarkably, ALA-treated animals presented higher mitotic index compared with untreated animals. These results suggest that ALA attenuates IRI of the rat liver in vivo with a reduction of cell death, whereas liver regeneration is increased [41].

### 2.4. ALA in Intestine

Intestinal IRI can complicate certain serious clinical conditions, including intestinal obstruction with strangulation, and intestinal transplantation [42,43,44]. In a rat model of intestinal IRI, Guven et al. showed that ALA decreased the intestinal injury [36]. Furthermore, the same effect was reproduced by Ebselen (2-phenyl-1,2-benzisoselenazol-3(2H)-one), a low-molecular-weight selenium compound, originally described as a drug that mimics the glutathione peroxidase (GPx) [45,46]. However, it was possible to observe that the combination of both drugs was much more effective decreasing lipid peroxidation products and increasing antioxidant enzymes than the administration of each drug alone [36].

### 2.5. ALA in Kidney

Rat renal IRI is a model that is extensively used to study acute kidney injury (AKI). In these animal models, it is possible to observe structural alterations in renal tubules, as well as impaired ions urinary concentration. Şehirli et al. showed that as well as in other organs ALA could reduce tissue damage in kidney IRI. This effect was mediated through reducing neutrophil infiltration, balancing the oxidant–anti-oxidant status and regulating the generation of inflammatory mediators [47]. Bae et al. showed that the treatment with ALA increased creatinine clearance compared with those in untreated rats. Also, ALA treatment reduced the degree of polyuria normalizing the excretion of sodium. The same authors described that ALA treatment attenuated the downregulation of aquaporins (AQPs) and sodium transporters in response to IRI [35].

### 2.6. ALA in Circulatory System

Myocardial ischemia-reperfusion is a major cause for the events of cardiovascular disease. Wang et al. investigated the protective effect of ALA against myocardial IRI and its mechanisms. They observed that myocardial IRI resulted in a significant increase of serum creatine kinase (CK), promoted oxidative stress and decreased the activities of antioxidant enzymes. In addition, apoptosis and inflammatory response were activated and aggravated in a time-dependent manner by IRI. All these alterations were attenuated by the administration of ALA before reperfusion [48].

He et al. tried to further explore the mechanisms underlying ALA’s cardio protective effect. In this sense, they observed, in a Langendorff model of IRI in rats that IRI led to cardiac dysfunction accompanied by an increase in products of phospholipid peroxidation. The pretreatment only with a high dose of ALA (5 × 10^−8^ M) improved these results. Moreover, ALA significantly up-regulated myocardial aldehyde dehydrogenase 2 (ALDH2) activity and these effects were reverted by its inhibitor. Similar results were achieved in vitro. It is interesting to remark that ALDH2 has been described to play a major role in detoxification of reactive aldehydes in a variety of organs [49]. This suggests that the cardioprotective effects of ALA on IRI are through a mechanism involving this enzyme activation and PKCε signaling pathways [37].

In line with He studies, Deng et al. described that ALA treated animals compared with untreated animals had lower tissue damage markers, smaller infarct size and less cell apoptosis and better cardiac functioning. Moreover, they showed that ALA pretreatment up-regulated Akt phosphorylation as well as Müller et al. described in liver. However, they noted an increase of nuclear factor erythroid 2–related factor 2 (Nrf2) nuclear translocation and hemoxigenase-1 (HO-1) protein levels in the myocardium, being this at least partially through activating PI3K/Akt pathway [38].

### 2.7. Summary of the Experimental IRI Models

Altogether, these IRI animal models, supports the concept that ROS could be an appropriate target to decrease tissue injury caused by the ischemia. Furthermore, ALA, through their pleiotropic mechanism of action may be a suitable candidate to reduce the deleterious effect of ROS. These animal models, also allow us to understand the putative molecular pathway involves in the effect of ALA on IRI, which includes the PI3K/Akt/Nrf2 pathways that controls the expression of genes involves in the detoxification and elimination of ROS and electrophilic agents. Although, it is probable that ALA effectiveness and mechanisms of action might vary depending on each tissue and organ where the ROS is produced. IRI is a process present in organ transplantation. However, these IRI animal models do not guarantee that the same pathway would be involved in organ transplantation, since the latter is a more complex process.

## 3. Clinical Trials

Beneficial effects of ALA treatment have also been observed in humans. Several studies have documented a positive therapeutic effect, particularly in diseases such as diabetes, atherosclerosis, neurodegenerative diseases, and AIDS, among others [26,50,51,52]. However, the most significant therapeutic effect of ALA is in diabetic polyneuropathy and cataract [53]. A four-year treatment using 600 mg ALA once daily in mild-to-moderate diabetic distal symmetric sensorimotor polyneuropathy resulted in a clinically meaningful improvement and prevention of progression of neuropathic impairments and was well tolerated by patients [54].

Standard oral dosages of ALA tend to be between 300–600 mg/daily and its administration is safe. There has not been defined an upper limit of ALA intake, but doses of 1800 mg/day caused no adverse effects over a 6–7-month period [55]. Moreover, in clinical trials, ALA administration has not caused severe adverse effects [56]. However, several common, mild and transient side effects were reported such as nausea, urticaria and itching, associated with high doses (1200–1800 mg/daily). Furthermore, in diabetic patients, mild hypoglycaemia was reported due to better cellular glucose uptake [57,58,59]. In 2014, it was informed a multiorgan failure and subsequent death within 24 h of a 14-year-old girl that ingested 6000 mg in a non-accidental intoxication [60].

Up to date there are two clinical trials published accomplished in humans suffering from IRI. First, Dünschede et al. studied the effects of preconditioning with ALA in twenty-four patients undergoing hepatic resection. In this study, aspartate transaminase (AST) and alanine transaminase (ALT) levels were significantly lower in ALA-pretreated group of patients (ALA: 600 mg i.v.; *n* = 12) compared with control group. Furthermore, the analysis of the biopsies showed histomorphological features of oncosis in control group but not ALA-treated patients. This result was confirmed in TUNEL assay. Therefore, the authors conclude that ALA reduced liver damage induced by the vascular occlusion and the liver resection [61].

In the second study, we analyzed the effect and safety of ALA administration in simultaneous kidney-pancreas transplant patients. It is worth to mention that ALA treatment was justified because all the patients included in the study suffered from diabetic polyneuropathy. Twenty-six simultaneous kidney-pancreas transplant patients were recruited for this preliminary study and grouped as follow: (i) untreated patients (*n* = 11), (ii) recipient treated with ALA just before the surgery (ALA: 600 mg i.v. *n* = 8); and (iii) donor treated just before the procurement and recipient prior to the transplantation (ALA: 600 mg i.v. *n* = 7). The primary outcome for this study was to evaluate the safety of the procedure by measuring the patient’s and graft’s survival. The secondary outcomes were to evaluate the inflammatory and biochemical markers and functional recovery of grafts. We observed that the treatment was safe since all patients and grafts survive three months after transplantation for both ALA-treated groups. Furthermore, the analysis of the results showed an effect of ALA treatment, particularly the group where both, donor and recipient were treated with ALA. This was statistically significant (*p* < 0.05) at the serum levels of proinflammatory cytokines (IL-8 and IL-6), alarmins immune mediators (Regenerating islet-derived protein 3 alpha, Reg-3a and secretory leukocyte protease inhibitor, SLPI) and amylase but not urea, creatinine and glucose. Moreover, we observed a tendency to a better kidney and pancreas clinical endpoints in donor and recipients ALA-treated groups. However, a higher number of patients should be recruited in order to confirm these preliminary results [62]. These results are extremely important since strengthen the idea that the generation of ROS plays a role in the pathophysiology of transplantation. Furthermore, and more importantly, it suggests that donor’s ROS affects the inflammatory status and integrity of the graft to be implanted. Therefore, we can speculate that a worthy therapeutic intervention against ROS, should start before the graft transplantation, probably in the donor and/or during the graft cold storage. Furthermore, preliminary results (40 patients recruited) suggest that a similar protective effect could be achieved with ALA administration in liver transplantation [63].

## 4. Discussion

The constant need to increase the number of donors made reconsider the criteria for organ donation, at least for kidney transplant. The old-for-old allocation policies or the inclusion of expanded criteria donors (ECD) are some of those efforts that try to decrease the high organ demand. The characteristics of these donors include an age higher than 60 years, or age between 50 and 59 years with at least two of the following features: history of hypertension, terminal serum creatinine >1.5 mg/dL, or cerebrovascular cause of death [64]. Currently, there is no doubt about the survival benefit of transplant patients with ECD over those that remain on dialysis. However, mostly of these criteria probably favor the production of ROS, diminishing the short and long term graft survival [65,66,67,68,69]. For example, the impact of age on ROS production has been described in humans and rats. The spontaneous ROS formation increases with age, in human neutrophils and rat cardiac tissues [70]. Besides age, there are others donor derived factors that may influence the transplant outcome, such as hypertension, obesity and cause of death. Higher ROS production was seen in hypertensive and obese subjects [71,72]. However, the donor death may boost the ROS production. In fact, it has been described an increase of ROS after brain death in rat kidney tissue [73]. In the same way, there are risk factors linked to the receptor. Those factors turn the recipient more susceptible to malfunction leading to a delayed graft function (DGF) [74]. This is a condition that is clinically defined as a need for dialysis in the immediate post-transplantation period and it has been related with graft loss [75].

In liver transplantation a post-reperfusion syndrome (PRS) could appear in the minutes after reperfusion. This PRS has been associated to IRI. The PRS is one of the causes of primary hepatic graft dysfunction, affecting the overall results of the transplant. As ROS being the main actors in IRI, treatment with an antioxidant such as ALA could be an adequate therapy and beneficial for the short- and long-term transplant outcome. Our preliminary result [63], treating the donor and receptor of liver transplant with ALA reinforced this issue.

**Figure 2:**
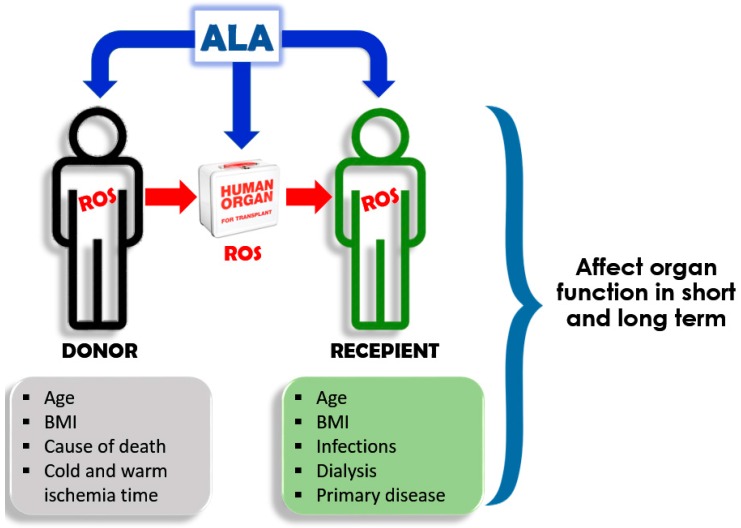
Putative targets for ALA in transplantation. ROS could be produced by the donor, the ischemic organ and the recipient during reperfusion. ALA could be administered to the donor and recipient, or could be used to perfuse the organ during cold ischemia time. (ROS, Reactive oxygen species; BMI, Body mass index; ALA, Alpha lipoic acid).

Overall, the experiments described above support the knowledge about the harmful consequences of ROS, as one of the main mechanisms responsible for IRI. However, the clinical efficacy of antioxidant therapies is questioned based on trials that showed lack of beneficial effects. For example, a randomized double-blind trial in kidney transplantation using another anti-oxidant (human recombinant superoxide dismutase) was not able to demonstrate benefit on serum creatinine and creatinine clearance at 48 h after surgery [76]. However, in another prospective randomized double-blind placebo-controlled trial, the same drug was able to reduce acute rejection episodes and to increase four-year graft survival [77]. In a further clinical trial, using the antioxidant *N*-acetylcysteine, the authors described less DGF and a better renal function at one year after transplantation [78]. The discrepancies among different trials could be due to the lack of certainty about the right dose and time lapses of drug administration related to the ischemia or reperfusion in transplantation. Furthermore, in relation to the donor and recipient we should identify which of them would be the best therapeutic target for antioxidant treatment. It is probable that the administration of antioxidants to a donor, slows the injury process inside of a cadaveric donor, resulting in a less damage graft with less proinflammatory characteristic. On the contrary, it is improbable that the administration of antioxidants turns to benefit if the organ is already damaged. Therefore, the efficacy of the administration of antioxidant to recipient will depend on the graft status prior the transplantation. Perhaps, the administration of the antioxidant to the donor could be difficult to achieve and approve. Therefore, another alternative could be the administration of ALA in the preservation solution in which organs are maintained for transplantation (Figure 2).

## 5. Future Prospect

The large number of preclinical studies mentioned above supporting the use of ALA in IRI treatment together with the protective results in preliminary human clinical trials would justify the use of ALA to reduce the impact of IRI and to improve the clinical outcome in organ transplantation. However, further precise and controlled randomized clinical trials, with a higher number of recruited patients and, ideally, multicenter, should be necessary to identify the best target, dose and time of ALA administration to improve the clinical outcomes in solid organ transplantation. These studies should be done in transplant recipient patients that we expect the worst short and long term clinical outcomes. For example, patients transplanted with organs derived from ECD or with high kidney donor profile index (KDPI). Although, ALA has shown safety, caution should be taken about putative interactions with immunosuppressive drugs used in the induction stage.

## Figures and Tables

**Table 1:** Summary of the effect of ALA treatment in ischemia reperfusion injury animal models.

Tissues and Organs	ALA Administration		*N*	Outcomes	References
	TIME SCHEDULE	DOSES			
Sciatic-tibial nerve	3 days before and 3 days after surgery	100 mg/kg/day intraperitoneal (i.p.)	44	Distal sensory conduction and fiber degeneration improvement in the short-time ischemia group	Mitsui et al. 1999 [31]
Ovary	21, 9 and 1 h before torsion of the ovary	36 mg/kg/day i.p.	32	Reduced tissue damage, MDA, NO and XO serum levels	Cosar et al. 2007 [32]
Testis	30 min prior to detorsion	100 mg/kg i.p.	35	Reduced testicular cell damage, apoptosis and MDA.	Ozbal et al. 2012 [33]
Liver	Ex vivo model: 20 min before ischemia	50 μM	15	Reduced LDH and PNP efflux, NF-κB and AP-1 activation and increased Akt phosphorylation	Müller et al. 2003 [34]
	In vivo model: 15 min before ischemia	500 μM i.v.	15	Reduced GST plasma levels and improved liver histology	
Kidney	48 and 24 h before ischemia and at 6 and 24 h after reperfusion	80 mg/kg i.p.	17	Increased creatinine clearance. Attenuated AQP downregulation and Na^+^ transporters. Reduced the polyuria normalizing the Na^+^ excretion	Bae et al. 2008 [35]
Intestine	1 day before and 3 days after surgery	10 mg/kg oral + ebselen (20 mg/kg) intragastrically	40	Increased SOD and GPx activity, reduce MDA and PCC levels and improved intestinal histology	Guven et al. 2008 [36]
Heart	*council house* model: 10 min before ischemia	Low dose: 10^−8^ M High dose: 5 × 10^−8^ M	42	High-dose treatment improved cardiac function, increased ALDH2 activity and decreased reactive aldehydes levels.	He et al. 2012 [37]
Heart	30 min before ischemia	15 mg/kg i.v.	120	Attenuated myocardial infarct size and preserved heart function. Up-regulated Akt phosphorylation and Nrf2 nuclear translocation. Increased expression of HO-1. PI3K inhibition abolished the beneficial effects.	Deng et al. 2013 [38]

MDA, malondialdehyde; NO, Nitric Oxide; XO, Xanthine Oxidase; LDH, Lactate dehydrogenase; PNP, Purine Nucleoside Phosphorylase; NF-κB, Nuclear Factor Kappa B; AP-1, Activator Protein-1; Akt, Protein Kinase B; GST, Glutathione S-transferase; AQP, Aquaporins; Na^+^, Sodium; SOD, Superoxide Dismutase; GPx, Glutathione Peroxidase; PCC, Protein Carbonyl Content; ALDH2, Aldehyde Dehydrogenase 2; Nrf2, Nuclear factor (erythroid-derived 2)-like 2; HO-1, Hemoxigenase-1; PI3K, phosphatidylinositol 3-kinase.

## Abbreviations

ALA	Alpha Lipoic Acid
LDH	Lactate Deshidrogenase
ROS	Reactive Oxygen Species
GST	Glutathione S-Transferase
ATP	Adenosine Triphosphate
TNF-α	Tumor Necrosis Factor Alpha
DHLA	Dihydrolipoic Acid
MDA	Malondialdehyde
SOD	Superoxide Dismutase
XO	Xanthine Oxidase
NO	Nitric Oxide
PNP	Purine Nucleoside Phosphorylase
NF-κB	Nuclear Factor Kappa B
AP-1	Activator Protein-1
GPx	Glutathione Peroxidase
CK	Creatine Kinase
ALDH2	Aldehyde Dehydrogenase 2
HO-1	Hemoxigenase-1
PCC	Protein Carbonyl Content
KDPI	Kidney Donor Profile Index
AST	Aspartate Transaminase
ALT	Alanine Transaminase
